# Defining and redefining harm reduction in the Lao context

**DOI:** 10.1186/1477-7517-9-28

**Published:** 2012-07-09

**Authors:** Vanphanom Sychareun, Visanou Hansana, Sysavanh Phommachanh, Vathsana Somphet, Phouthong Phommavongsa, Brigitte Tenni, Timothy Moore, Nick Crofts

**Affiliations:** 1 Faculty of Postgraduate Studies, University of Health Sciences, Vientiane, Lao PDR; 2 Nossal Institute for Global Health, University of Melbourne, Melbourne, Australia; 3 Melbourne School of Population Health, University of Melbourne, 207 Bouverie Street, Parkville, 3100, Melbourne, Australia

## Abstract

The response to drug use in Laos has focused on reducing opium supply (supply reduction) and rates of drug use (demand reduction). However, recently there is increased interest among government counterparts to discuss and develop broader responses to injecting drug use (IDU) including the introduction of harm reduction programs. The concept of harm reduction has just been introduced to Lao PDR and as yet there is no agreement on a definition of the concept. We highlight here a range of issues that remain controversial in Lao PDR in the HIV, drug use and harm reduction discourse, the definition of 'harm reduction' and related terms; and the scope of harm reduction.

This was a qualitative study, consisting of in-depth interviews with 27 law enforcement and 8 health officers who work in the fields of HIV and/or drug control about their understanding of HIV related to drug use, and concepts of harm reduction. Content analysis was performed to identify the coding, categories and themes.

We found that law enforcement officers in particular had limited understanding about harm reduction and the feasibility and appropriateness of harm reduction services in the Lao context.

Harm reduction should be a core element of a public health response to HIV where drug use and IDU exists. Recommendations include the necessity of increasing the awareness of harm reduction among law enforcement officers and providing appropriate evidence to support the needs of harm reduction policy and programs. HIV prevention and treatment strategies should be integrated within existing social and cultural frameworks, working with the task force for HIV/IDU and other government counterparts.

## Background

### Illegal drugs in Lao PDR

The use of illicit drugs is growing throughout South East Asia, including Lao PDR, with negative implications for individuals and public health becoming increasingly apparent [1]. The prevalence of illegal drug use in Lao PDR is not known with any accuracy, but it has been estimated to be higher than 2% [2]. The major substances consumed recreationally in Lao PDR are opium and its derivatives, amphetamine type stimulants (ATS) and volatile substances (glue, petrol). It was estimated in 2004 that there were 8,000 injecting drug users (IDUs) in Lao PDR [3]. A school survey in 1999 found 17% of youth had tried some form of drugs in their life and 7% had used marijuana, hashish, inhalants or amphetamines [4].

The prevalence of amphetamine use was 0.7% of the population aged 15–64 years, while opiate use was estimated at 0.5% of 15–64 year olds [5] leading to an assumption that approximately 40,600 people use ATS and 29,000 use opium. Several small studies into ATS use in Laos initiated by the Lao PDR government have focused on the prevalence of drug use among vulnerable groups, and previous quantitative studies on drug use in Northern Laos [6,7]. Other surveys conducted by UNODC in 2002 suggested high ATS lifetime prevalence for unemployed youth (42%), bar clients (34%), and service women (14%) (LCDC/UNODC/, [8-10]).

Laos shares borders with areas of high prevalence of IDU, especially southern China and Vietnam, and lies on a heroin trafficking route [11]. Heroin has been found in border areas, in particular along the Lao-Vietnam border in the north-east*,* where heroin is cheaper than opium, facilitating the transition of former opium users to heroin [12]. A survey carried out by LCDC/UNODC found 2.8% of the population 15 years old and above in 35 villages in 3 border areas were using heroin [13], and evidence of transitions from opium to heroin use due to the decline in opium production and the consequent decrease in its availability [5].

### HIV and IDU in Lao PDR

Because IDUs are vulnerable to infection with HIV, and have emerged in some communities in the border areas, there is a need to plan preventive responses; however, there is no systematic collection of data relating to IDUs [6]. A straw in the wind may be indicated by the results of a recent rapid assessment and response exercise (RAR) carried out in two Northern provinces, which found 1.7% of 530 drug users surveyed were HIV positive [14].

HIV is therefore emerging as a potentially serious health issue for Lao PDR, even though currently Laos is categorized as a low HIV prevalence country, with an estimated 0.2% HIV prevalence [15]. According to a UNODC HIV Report [3], 2% of the estimated 1,400 HIV cases in Lao PDR acquired the infection through IDU. This trend could potentially change if drug consuming patterns shift [16]. Higher HIV prevalence rates have however been found among sex workers at 1.1% in 2001 (CHAS/FHI 2001 [17]) and increased to 3.3% in 2004 [18] and the prevalence among men who have sex with men (MSM) in Vientiane was 5.6% [19,20].

### Harm reduction in Lao PDR

Recently, with UN support, the government of Lao PDR set up a ‘Task Force on HIV and Drug Use’ with the objectives of developing policy, proposing concrete activities to address the vulnerability of drug users to HIV, and developing guidelines for harm reduction and treatment of drug use [21]. This Task Force is jointly chaired by the Lao PDR National Commission for Drug Control and Supervision (LCDC) and the Ministry of Health, and is composed of different stakeholders enlisting many different sectors in the response to HIV.

Harm reduction for IDUs is entrenched in the National HIV/AIDS/STI Strategy 2011–2015 [22] which includes measures to increase awareness of the vulnerability of drug users, to ensure an enabling legal and policy environment for harm reduction, and to expand behavioural change interventions including safe injecting. The strategy includes the following expected outcomes:

(i) at least 55% of injecting drug users will use sterile injecting equipment

(ii) 60% of drug users are reached through harm reduction interventions;

(iii) 55% of IDU report consistent condom use with their sexual partners.

So far the harm reduction activities are very incipient in the country and rely on outreach work. HIV/AIDS Law was launched in July 2010 which included harm reduction for IDU in Article 16 [23]. Despite the inclusion of harm reduction in the National HIV Strategy, there are no policies to support the delivery of harm reduction services, and there are not yet any harm reduction services other than information and counselling for IDUs. The Government of Laos and donor agencies have recently agreed to implement harm reduction programs in Laos despite the lack of data to inform these programs.

Harm reduction themed programs to date have included the SIDA supported ‘Harm Reduction, Human Rights, Human Resources’ (HR3) Project from 2008 to 2009, the objectives of which were to build regional capacity to deliver harm reduction services to prevent and reduce HIV related harm among IDUs. The activities of the project included identifying stakeholders and training themes and advocacy and technical support for the development, operation, and extension of community based delivery services and a combination of local and regional training in order to understand the requirements and conditions for success of harm reduction programs and a survey of IDUs in the Northern provinces which was co-funded by SIDA and HAARP [24].

A more recent harm reduction initiative is AusAID’s HIV/AIDS Asia Regional Program (HAARP), which expanded to include Laos in October 2009. Its primary objective is to reduce HIV transmission associated with drug use, including IDU in Lao PDR. Expected outcomes of the program include strengthening the National Task Force on HIV and Drug Use, establishing Pilot Provincial Task Forces in 2 provinces, developing evidence and data to understand the level of coverage and types of services provided, developing the capacity of harm reduction service providers, and strengthening the capacity of high level officials in harm reduction among the law enforcement sector and drug treatment personnel [25]. The Burnet Institute also carried out training on harm reduction among law enforcement sectors in 2009. In addition, activities implemented in the HAARP project in Laos also included harm reduction training for law enforcement and study visits to observe harm reduction service delivery in other countries.

## Methods

This study employed qualitative research methods to interview key informants from the law enforcement sector such as the Laos Committee on Drug Control (LCDC), Provincial Committee on Drug Control (PCDC) and police officers and members of the health sector such as the Centre for HIV/AIDS/STI (CHAS) and staff of drug rehabilitation centres. Additionally, staff from relevant UN agencies such as WHO, UNAIDS, and UNODC were interviewed. Harm reduction program documents and reports relating to ATS were reviewed, and the investigators informally spoke with various agencies to understand how the definition of harm reduction in Laos is constructed. In-depth interviews were conducted to explore personal narratives and experiences related to drugs, knowledge of harm reduction program aims and approaches, and perceptions of HIV and drug use. In all, 27 law enforcement officers and 8 health officers currently working with HIV and drug control and supervision were interviewed about their understanding about HIV related to drug use and concepts of harm reduction.

The research protocol, field guides and consent forms were reviewed and approved by the National Ethical Committee for the Health Research, Ministry of Health, Lao PDR and ethics approval was obtained from the University of Melbourne. Verbal consent was obtained from the participants after the research team had explained the objectives using the Plain Language Statement of the study and expectations of the study participants. Study participants were assured confidentiality and privacy.

### Study setting

The study was undertaken in Vientiane, the largest city in Lao PDR with a population of 700,000. Bordering north-east Thailand, 82% of the province of Vientiane Capital’s population lives in urban areas. In contrast, 25% of the total Lao population lives in urban areas [26]. Government, UN and nongovernment agencies are concentrated in Vientiane Capital.

### Data analysis

Content analysis was carried out using both manifest and latent analysis. The interviews and discussions were taped, transcribed, summarised and translated into English by the interviewers and the researchers. A transcript-based analysis was used for the project with the aid of Nvivo software. A preliminary reading of the transcripts identified potential themes and/or patterns. Quotes were identified that exemplified or reflected these themes. Emergent themes included the understanding of harm reduction, components of harm reduction, attitudes towards harm reduction and understanding of the role of law enforcement in supporting implementation of harm reduction in Laos.

## Results

### Understanding of harm reduction

Most key informants at the central level from the law enforcement and health sectors had heard of harm reduction, however, they showed little understanding of the definition and components of harm reduction approaches. Harm reduction as a concept had only been introduced in Lao PDR a few years prior by the HR3 and HAARP projects. Many key informants mentioned that harm reduction is a new concept for Laos:

"“Harm Reduction concepts are new for Laos and we cannot move things so fast and you cannot change things faster. This is a testing time” (Male, 40 years old)."

There was little agreement amongst the key informants at the central level about the definition of ham reduction. Some key informants from LCDC and MOH and MoPSc, who have been trained in harm reduction, could give some definition of harm reduction as demonstrated by:

"“Definition of harm reduction encompasses reducing the severity of IDUs” (Female, 59 years)"

Some NGO staff gave a definition of harm reduction that encompassed a broader, more philosophical understanding of the harm reduction approach that was more in keeping with the international standard.

"“The definition of harm reduction of drug use is a policy determination, planning, service and an operational activity for health risk reduction, socio and economically related to drug use” (Female, 55 years old)"

"“Harm reduction (HR) concept is defined as reduced harm of the IDUs by exchanging clean needles” (Group interview)."

"“Harm reduction concept is denoted that drug users still use drugs, but only to avoid HIV/AIDS” (Male, 51 years old)"

Some key informants mentioned that the definition of harm reduction should be broader and encompass drug users (DUs) in addition to IDUs, and should range from the prevention of drug use to the prevention of the transition from non-injecting to injecting forms of drug use.

"“I think that non-injecting drugs are widespread, why we did not discuss about HR [harm reduction] related to DUs, while IDUs are not widespread yet” (Male, 35 years old)."

Some respondents mentioned that harm reduction should also encompass all drugs and this was seen as one of the strengths of the approach.

"“HR is not just for IDUs, this should also include DUs such as ATS, providing knowledge on HIV/AIDS and providing a means of prevention of HIV/AIDS” (Female, 54 years old)."

"“HR should cover both DUs and IDUs while the pattern of drug use in the country is mainly amphetamine type stimulants. There was a lot of amphetamine stimulant users compared to IDUs, so the HR should be targeted to DUs as well as IDUs.” (Male, 56 years old)"

Some respondents highlighted the problem of translating “harm reduction” into the Lao language to capture its true meaning. There was no one word in Lao for “harm reduction” which was likely to create confusion in the Lao context.

"“The definition of HR has not been cleared yet due to various translations, so there are different meanings. Hence we have to discuss this, using the medical term would be easier” (Male, 55 years old)."

Most of the law enforcement respondents at the grassroots level did not understand the concept of harm reduction and confused it with supply reduction. When asked about harm reduction, they referred to the establishment of drug free villages and villages without criminal offences:

"“We have a policy to create villages without drugs based on four basic standards (no drug users, no drug dealers, no drug production and no persons hiding drug users) and 11 activities. Villages without drugs does not mean that they do not have drugs in their villages, however, it means that they have to reduce drugs step by step and meet the criteria mentioned above by at least 90%. Villages without drugs are declared year by year” (Male, 35 years old)."

"“Now, we are establishing safe villages without crimilas and drug free villages. Our village is a public security village which includes 5 criteria such as village health model, cultural village, village without criminal cases and drug free villages.…(Male, 56 years old)"

"“For harm reduction, I did not understand clearly. I am responsible for VTE capital city, so I did not have activities to cut the trafficking routes of transportation of drugs. I just provide health education or propaganda to drug users. If we can reduce drug use, we also could reduce drug dealers” (Group interview)"

Many respondents maintained that Laos did not have IDU or that there was no evidence of HIV amongst the small number of IDU in Laos. Many believed that one implemented harm reduction programs only if there was a high prevalence of HIV amongst the IDU population and referred to the Australian experience:

"“Many think you only implement when you already have problem of HIV in IDUs. – like in Australia. Our country has many problems such as poverty and high maternal mortality” (Male, 63 years old)"

"“The IDUs are not the priority yet as the prevalence of IDUs is small, there was some evidence of IDUs in the 2 northern provinces, thus there will be some HIV/AIDS responses” (Male, 73 years old)"

"“We did rapid assessment of IDUs in the 2 Northern provinces. Among 550 DU/IDUs, 49 were IDUs and the prevalence of HIV among DU and IDUs is 1.5%, however, we could not say this is representative because the sample size is small.” (Female, 62 years old)"

Many respondents also thought DU and IDU were at risk of HIV due to sexual transmission rather than blood borne HIV risk from using unsterile injecting equipment. They therefore did not see the need for harm reduction programs.

"“I did not agree that we have to focus on IDU because the problem of IDU is still low, so we need to focus on prevention. The transmission of HIV/AIDS in our country is transmitted mainly through heterosexual intercourse. So, we need to pay attention to this issue” (Male, 63 years old)"

### Components of harm reduction

Most respondents who mentioned harm reduction programs in Laos were focused on just one or two elements of the comprehensive strategy – needle and syringe programs (NSP) and opioid substitution therapy (OST); and even these two elements were incomplete. Other components considered being integral to a comprehensive harm reduction approach such as peer outreach, condom distribution and ARV provision were mentioned only by a few respondents.

"“....The component of HR consisted of different components such as methadone, NSP, condom distribution and providing health education to drug users” (Female, 55 years)"

Few of them highlighted the overall the importance of a comprehensive harm reduction approach:

"“The components of HR are the same as in anywhere else in the world which consists of health education, condom distribution, syringe and needle exchange and methadone” (Male, 40 years old)."

There was disagreement about the essential elements of a comprehensive harm reduction program. Some respondents’ answers focused on peer education, outreach programs and condom use. No respondents could give details of all nine components of a comprehensive harm reduction program and often omitted the diagnosis and treatment of and vaccination for hepatitis and the prevention, diagnosis and treatment of tuberculosis (TB).

"“The components of HR consist of different components such as methadone, NSP, condom distribution and providing health education.”(Male, 54 years old)"

"“We need to discuss the HR components in our country, which one is suitable for Laos?” (Male, 55 years old)"

### Attitudes towards harm reduction

Most key informants hold negative attitudes towards the introduction of harm reduction and saw it as promoting the use of new prohibited drugs. They saw it as inappropriate for the Laos context and against the law:

"“If we introduced HR programs into Laos, this in turn will promote IDUs and demonstrate that this is more effective than DU and the effects last longer than DUs” (Male, 60 years old)."

"“I think that the distribution of needles is to promote them to use by IDUs because they would like to try new things. Right now, they used to swallow or inhale. When they have needles, they would like to try it” (Male, 38 years old)."

"“HR program is not appropriate in our context, because it will be more encouragement, and it would be against the law” (Male, 54 years old)."

"“If we introduced HR related to IDUs, this seemed to me that we encouraged them to use IDUs as they would like to try injecting” (Male, 35 years old)"

Respondents’ perspectives also reflected the low priority of IDU given Laos’ many competing public health priorities and limited financial resources to implement harm reduction.

"“We could not afford to buy methadone for a small proportion of IDUs. If we have a natural disaster, how can we find the budget to buy methadone. The government has not enough budget for the salary of government staff. If we spend our budget to buy methadone, it is better to spend that budget to increase the staff’s salary. So, it is better to do prevention, reduce drug supply by arresting drug smugglers and fine them. Then, we can use that money to help the remote people with regard to vocational training. For the developed countries, the government has budget to provide methadone. For example, harm reduction in Vietnam is internationally funded, what happens when international aid in Viet Nam finishes?” (Male, 70 years old)"

Lack of evidence of IDUs was also mentioned as a reason not to implement harm reduction programs for IDUs. Respondents highlighted the need to know more about the size of the IDU population before harm reduction programs could be initiated.

"“The government needs some evidence about the size of IDUs, what are the negative health effects of IDUs, what percentage of them need assistance? What percentage have HIV/AIDS, what percentage have HIV from sexual intercourse, what percentage have HIV from DUs and IDUs. Then, we can discuss HR such as NSP and methadone” (Male, 70 years old)"

The issue of opioid substitution therapy such as methadone was raised by law enforcement and those from the health sectors at the central level. The attitude toward methadone substitution therapy was rather controversial among different groups. Some of the LCDC and health officers considered opium tincture therapy to be substitution therapy and a necessary element of a harm reduction strategy. Respondents working in the field of drug control and supervision considered methadone substitution therapy to be absolutely unacceptable. In contrast, a few respondents from NGOs stated that substitution therapy could be very helpful in specific cases:

"“I think that providing methadone is a good way because it is able to help drug users to change from illegal drug use to legal, and not develop to be IDUs, and the IDUs will be able to check their health status. Thus, IDUs can access health services and the health care providers can provide health education to IDUs.” (Female, 37 years old)."

Few of them mentioned the possibility of introducing methadone therapy to help IDUs and how the advantages of methadone far outweighed the disadvantages.

"“....The disadvantage is that we support them to use drugs; however, if drug users could not withdraw from drugs, it is better to switch from illegal use to legal use and we can manage them. If we think about introducing methadone, we need to get international support as our GPD is small and the sustainability is still a problem for government. When we get international support from the beginning, then when we can stand by ourselves, so the international aid will be stopped step by step. For example, ARV treatment is also expensive, however, the government received international support for ARV” (Female, 52 years old)."

### Understanding of the role of law enforcement in supporting implementation of harm reduction

Some key informants mentioned that there is a need to have law enforcement support for harm reduction programs.

"“If you were really wanted to implement harm reduction, you need to change the law or you need to make a new law to authorize HR intervention” (Male, 44 years old)."

"“We need to discuss with LE to understand HR because this is wrong according to LE, distributing needles are illegal because they use drugs illegally”. (Male, 44 years old)."

Some law enforcement saw harm reduction as an essential component to drug control and complementary to supply and demand reduction.

"“In reality, drug activities should incorporate three components, such as, drug demand reduction, supply reduction, and harm reduction.” (Male, 45 years old)"

Despite the significant role law enforcement plays in supporting harm reduction programs, many respondents stated that law enforcement officers and the government did not understand or accept harm reduction yet and therefore suggested that there is a need advocate for harm reduction to the policy makers and law enforcement officers.

"“We need to advocate to the government of the need for HR. If we don’t implement HR, there will be spread of HIV/AIDS and increases in the cost of treatment of HIV/AIDS even though right now, the government does not accept it, they will accept it in the future. We need to provide some evidence to them about the cost of treatment if there was no intervention and how the cost will be reduced if there was some interventions” (Female, 52 years old)."

One respondent said that it was necessary to train police in harm reduction so that they better understand and support programs:

"“In order to implement harm reduction program, firstly we have to advocate to the police for their understanding about harm reduction, and then train the police as trainers how to advocate about HR for the other police groups. This is because police are more likely trust each other and are better at providing information than others” (Female, 37 years old)."

## Discussion

Considerable disagreement and debate exists within Laos about the definition and the essential components of a harm reduction program, and about harm reduction’s relevance to the Laos context. Many stakeholders believe harm reduction programs are not appropriate for Laos, but for differing reasons, and without necessarily a fully informed appreciation of what such an approach entails. It was clear that many police in Laos did not understand harm reduction, however this is understandable given that its relevance or acceptance has never been articulated from a police point of view.

The lack of national data on prevalence of injecting drug use is often cited as reason to delay the introduction of harm reduction programs until there is reliable data and evidence. In addition many stakeholders believe that harm reduction programs should only be initiated when there is evidence of a high prevalence of HIV within IDU populations – when in reality harm reduction program need to be initiated *early*, to prevent the rapid spread of HIV among people who inject drugs. This reluctance could reflect uninformed attitudes, or the lack of understanding of or belief in the aims and evidence base on which harm reduction approaches are built, or negative and prejudiced opinions towards drug users. District police in particular were confused as to what harm reduction comprises and in many instances described and expressed a preference for a supply reduction approach, despite the ineffectiveness of uni-dimensional supply reduction approaches in diminishing increased supply and demand of illegal drugs.

Other members of law enforcement were sceptical of the benefits of harm reduction, especially needle syringe programs, and saw it as encouraging, condoning and potentially leading to increases in injecting drug use, especially in a context of low rates of IDUs. This common misconception can easily be refuted by data from harm reduction programs implemented in a vast array of culturally, religiously and politically diverse contexts around the world – including countries very close to Laos, such as Vietnam and Malaysia.

Most respondents pointed to the limitations of applying a harm reduction approach only to injecting drug use and not to non-injecting drug use, and drew a link between non-injecting drug use and the risk of sexual transmission of HIV. These are valid points given Laos’ much higher rates of ATS and traditional opium use and the focus of current programming on harm reduction for injecting drug use only. This gap highlights the challenges Laos faces to developing comprehensive and responsible drug policy given its history, geography and patterns of drug use. The response to drug use needs to anticipate an increase in injection, especially in the border regions and interventions need to reflect the local context but also be mindful of evidence from other countries.

The confusion in terminology and understanding highlights the need for clear and consistent harm reduction messaging and strong leadership at all levels of government, health and law enforcement. Countries in the Mekong region are facing multiple drug-related challenges, most especially major epidemics of amphetamine use, most not injected. This poses the further challenge for the public health and harm reduction communities to broaden their focus to include all aspects of drug use, and thus respond both to the communities’ perceived needs (harms associated with amphetamine use, especially sexual transmission of HIV and transitions to injecting) and those known to be potentially threatening Laos (heroin injecting and HIV transmission).

## Conclusion

This paper provides a platform to explore the various understandings of harm reduction as it applies to the Lao context, and how various stakeholders inform this understanding. It highlights the need for a mutual understanding among programmers, government, health sector and law enforcement of the basic definition of harm reduction, its’ essential components and what harm reduction aims to achieve. Additionally, it underscores the need for police advocates to take the lead in framing harm reduction from a law enforcement perspective if harm reduction and humane and effective drug policy in Laos is to progress. This will help to ensure that future harm reduction programs are contextually relevant, based on solid evidence and incorporate a multi-sectoral approach.

## Competing interests

The authors declare that they have no competing interests.

## Authors' contribution

VS, SP, VS and PP collected data. VS, BT, NC collated and analysed the data. VS, VH, BT, TM and NC drafted the manuscript. All authors read and approved the final manuscript.
